# Research on Elastic–Plastic Contact Behavior of Hemisphere Flattened by a Rigid Flat

**DOI:** 10.3390/ma15134527

**Published:** 2022-06-27

**Authors:** Wangyang Zhang, Jian Chen, Chenglong Wang, Di Liu, Linbo Zhu

**Affiliations:** 1 School of Mechanical and Electrical Engineering, Xi’an Polytechnic University, Xi’an 710048, China; wangyangzhang20@163.com (W.Z.); wangchenglong123@126.com (C.W.); ldljb406@163.com (D.L.); 2 School of Chemical Engineering and Technology, Xi’an Jiaotong University, Xi’an 710049, China; linbozhu@mail.xjtu.edu.cn

**Keywords:** flattening contact behavior, elastic–plastic range, elastic–plastic constitutive model, finite element method

## Abstract

The contact behavior of a hemisphere pressed by a rigid plane is of great significance to the study of friction, wear, and conduction between two rough surfaces. A flattening contact behavior of an elastic–perfectly plastic hemisphere pressed by a rigid flat is researched by using the finite element method in this paper. This behavior, influenced by different elastic moduli, Poisson’s ratios, and yield strengths, is compared and analyzed in a large range of interference values, which have not been considered by previous models. The boundaries of purely elastic, elastic–plastic, and fully plastic deformation regions are given according to the interference, maximum mean contact pressure, Poisson’s ratio, and elastic modulus to yield strength ratio. Then, a new elastic–plastic constitutive model is proposed to predict the contact area and load in the elastic–plastic range. Compared with previous models and experiments, the rationality of the present model is verified. The study can be applied directly to the contact between a single sphere and a plane. In addition, the sphere contact can also be used to simulate the contact of single asperity on rough surfaces, so the present proposed model can be used to further study the contact characteristics of rough surfaces.

## 1. Introduction

The elastic–plastic contact behavior between hemisphere and rigid plane is one of the fundamental problems in particle mechanics [1,2], contact mechanics [3,4,5,6,7,8,9], and biomechanics [10]. Mechanical surfaces are microscopically rough. Contact between two rough surfaces can be equivalent to contact between a hemisphere and a rigid flat [11,12,13]. The study of elastic–plastic contact behavior between hemisphere and a rigid flat is of great significance to the analysis of electrical contact [14,15,16,17], friction [18,19], and wear [20,21] between two rough surfaces. 

The early contact model only predicted the elastic contact behavior between the hemisphere and the rigid flat. Greenwood and Williamson (GW model) [22] analyzed the pure elastic contact behavior between hemisphere and rigid flat. With the increase of interference, the hemispheres yield, and the contact state changes from pure elastic to elastic–plastic. Based on the Hertz solution [23], the GW model presented the critical normal interference (*ω_c_*), critical contact load (*F_c_*), and critical contact area (*A_c_*) formulas for the initial yield of the hemisphere. However, the Poisson’s ratio (*ν*) effect is ignored in their predicted formulas for these critical contact parameters. Later, Lin and Lin (LL model) [24] and Chang et al. (CEB model) [25,26] improved the GW model and further considered the effect of different *ν* on these critical contact parameters. In the elastic–plastic contact stage, the contact behavior is highly nonlinear due to the existence of plasticity. Some scholars have predicted the elastic–plastic contact behavior between hemispheres and rigid planes through experiments [27,28]. However, the experimental results only apply to specific materials. Because the finite element method (FEM) can accurately simulate the contact process between hemisphere and rigid flat of different materials, some researchers have analyzed on the elastic–plastic contact behavior between hemisphere and rigid flat based on the FEM [29,30,31,32,33]. 

Kogut and Etsion (KE Model) [31] analyzed the effect of the ratio of the elastic modulus to yield strength (*E*/*Y*) on the elastic–plastic contact behavior in the range of 100 to 1000 based on FEM. They demonstrated that when the dimensionless mean contact pressure (*p*/*Y*) reaches its maximum, the contact state changes from elastic–plastic to fully plastic, with the corresponding dimensionless interference (*ω*/*ω_c_*) as a constant value equal to 110. Based on the analysis results, they presented the empirical relationship between the *p*/*Y* ratio, dimensionless contact load (*F/F_c_*), dimensionless contact area (*A/A_c_*), and **ω*/*ω_c_** within *ω*/*ω_c_* = 110. By analyzing the hemisphere and rigid plane contact under different *Y* based on the FEM, Jackson and Green (JG model) [32,33] proposed that the maximum value of *p/Y* was not the constant value equal to 2.8, which had been predicted by Tabor [34] for different *Y*. Based on the results, they presented a new model for predicting the elastic–plastic contact parameters. They defined the initial range of elastic–plastic; however, the termination range of elastic–plastic is not clear in their research. Quicksall et al. [35] used FEM to study the elastic–plastic contact behavior of malleable cast iron, aluminum, titanium, copper, and bronze hemispheres with rigid planes. The results were compared with the predicted by the KE and JG models. They showed that the prediction accuracy of the JG model is higher than that of the KE model, but the formula of the JG model is more complex than that of the KE model. 

Elastic–plastic is a complex nonlinear behavior. For the large *ω*/*ω_c_* values and different materials, the previous results of fitting may not be accurate enough for small interference or specific materials. Based on the KE and JG models, many scholars analyzed the effect of material properties and contact properties on hemispheric elastic–plastic contact behavior [36,37,38,39]. Brizmer et al. (BK model) [37] analyzed the effect of material properties and contact conditions at the end of elastic deformation in spherical contact. They found that the initial yield of plastic materials always occurred at a point on the axis of symmetry of the hemisphere, while the brittle failure always occurred at the hemisphere contact surface. Shankar and Mayuram (SM model) [38] analyzed the contact behavior between hemisphere and rigid flat under different *Y* and compared the results with those predicted by KE and JG models. They found that when *E*/*Y* is 79.4, the prediction accuracy of previous KE and JG models is poor. Therefore, they improved the JG and KE models and proposed a new hemispheric contact model. Then, they further considered the effect of the tangential modulus (*E_t_*) on hemispheric contact behavior [39]. Malayalamurti and Marappan (MM model) [40] analyzed the influence of different hemispherical radii (*R*) and *Y* on hemispherical elastic–plastic contact behavior. Based on the analysis results, they proposed an empirical relationship between *F*/*F_c_*, *A*/*A_c_*, and *ω*/*ω_c_*. Sahoo and Chatterjee (SC Model) [41,42] used FEM to analyze the elastic–plastic contact behavior between a sphere and a rigid flat under different *E*, *E_t_*, and *R*. They observed that the hemispheric elastic–plastic contact behavior is similar at different *R*. They demonstrated that when *E_t_* is small, the result is similar to these predicted of the KE model. With the increase of *E_t_*, the prediction accuracy of the KE model becomes worse. However, they do not provide a prediction formula. Under different *Y* and *ν*, Megalingam and Mayuram (MM model [43]) analyzed the elastic–plastic contact behavior between hemisphere and rigid flat. Then, the effect of *E_t_* on the elastic–plastic contact behavior was further considered [44]. They showed that *Y* and *E_t_* have a greater effect on hemispheric elastic–plastic contact behavior than *ν*. They also presented a formula for calculating *F*/*F_c_* and *A*/*A_c_* in the elastic–plastic range. Recently, Gheadnia et al. [45,46,47] analyzed elastic–plastic contact behavior between the deformable hemisphere and the flat using FEM by controlling the yield strength ratio between hemisphere and flat (*Y*_1_/*Y*_2_). They demonstrated that the elastic–plastic contact behavior is related to the *Y*_1_/*Y*_2_ ratio. 

Due to the complexity of elastic–plastic problems, there is still no closed solution. Although some hemispheric contact models exist, there is still a lack of a model to compare and analyze hemispheric elastic–plastic contact behavior over different *E*, *ν*, *Y*, and large *ω*/*ω_c_* values. In this paper, the elastic–plastic contact behavior of the hemisphere pressed by the rigid flat by *E*, *ν*, and *Y* was analyzed based on FEM. According to *ω*/*ω_c_*, maximum *p*/*Y*, *ν*, and *E*/*Y*, the boundaries of elastic, elastic–plastic, and fully plastic deformation regimes were given. A new elastic–plastic constitutive model was proposed to predict the *A*/*A_c_* and *F*/*F_c_* in the elastic–plastic range. Compared with the results predicted by previous models and experiments, the rationality of the present model is verified. In Section 2, the critical contact parameters for the initial yield of hemispheres are analyzed. The FE model is presented in Section 3. In Section 4, the empirical formula of hemispheric elastic–plastic contact is demonstrated by curve fitting, and the results are compared with previous models. The conclusions are presented in the last section. 

## 2. Critical Formula

**Figure 1:**
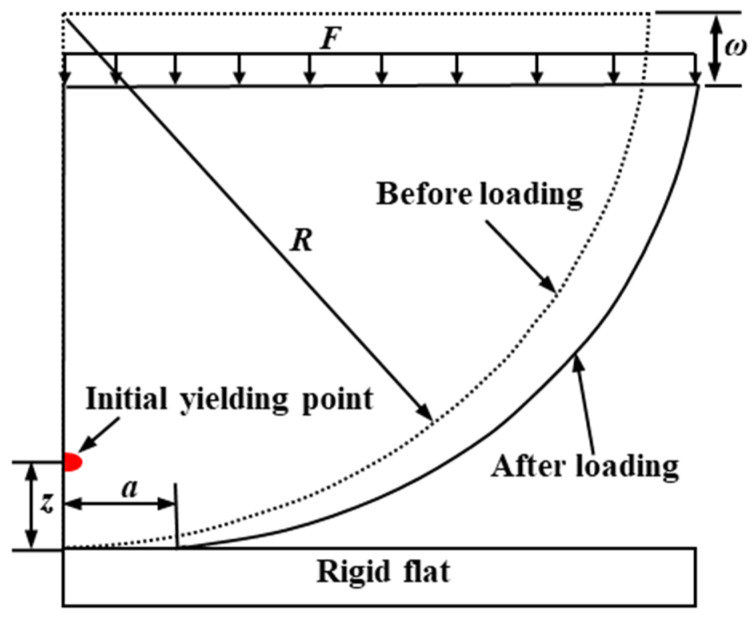
A flattening model before (dashed line) and after (solid line) loading.

A flattening model of a deformable hemisphere pressed by a rigid flat before and after loading is shown in Figure 1. A uniform downward load (*F*) is applied to the top of the hemispheres to simulate loading. At a small interference (*ω*), the contact state is purely elastic. With an increase of interference, the initial yield occurs at the contact subsurface depth *z* of the hemisphere, marking the end of purely elastic deformation or the inception of elastic–plastic deformation. 

The interference (*ω*) at the initial yield is called the critical interference, *ω_c_*, which is calculated according to the formula provided by Johnson [3] and given by

(1) $$\omega_{c}={\left(\frac{\pi p_{0}}{2E}\right)}^{2}R,$$

(2) $$\frac{1}{E}=\frac{1-\nu_{1}{}^{2}}{E_{1}}+\frac{1-\nu_{2}{}^{2}}{E_{2}},$$

(3) $$\frac{1}{R}=\frac{1}{R_{1}}+\frac{1}{R_{2}},$$

where *E* is the equivalent elastic modulus and *R* is the equivalent radius. *ν*_1_, *ν*_2_, and *E*_1_, *E*_2_ are the Poisson’s ratios and elastic moduli of the two materials in contact, respectively. *R*_1_ and *R*_2_ are the radii of the contact pairs. *p*_0_ is maximum Hertzian pressure, which is listed in Table 1.

**Figure 2:**
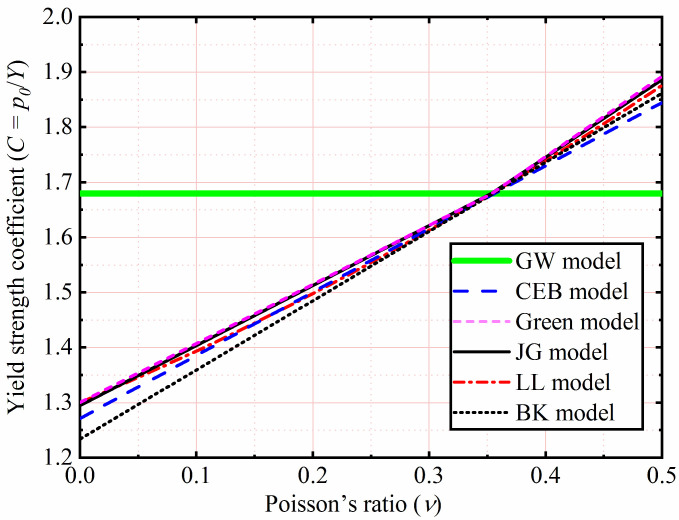
Yield strength coefficient as a function of Poisson’s ratio.

As shown in Table 1, *p*_0_ is related to material hardness (*H*), where *H* is equal to 2.8 *Y* predicted by Tabor [34] in the GW model [22], CEB model [26], and LL model [24]. However, this relationship between *H* and *Y*, recently pointed out by the JG model [33] and SM model [38], was not the constant value equal to 2.8. In the Green model [32], BK model [37], and JG model, *p*_0_ is predicted by using the von Mises yield criterion and the stress field of Johnson, which seems more reasonable than the GW model, CEB model, and LL model. The yield strength coefficient (*C*) is a function of Poisson’s ratio (*ν*). It is shown in Figure 2. The critical results of all models are the same at *ν* = 0.35. For the common materials (0.2 ≤ *ν* ≤ 0.45), except for the GW model, the critical interference of the JG model, CEB model, Green model, LL model, and BK model are similar. 

Considering the above discussion and the meaning of *p*_0_, the critical interference is chosen in this study by the JG model and given by

(4) $$\omega_{c}={\left(\frac{\pi CY}{2E}\right)}^{2}R.$$

The contact state is purely elastic before the initial yielding of the hemispherical contact (*ω* < *ω_c_*). The contact load (*F_e_*) and the contact area (*A_e_*) can be expressed as

(5) $$F_{e}=\frac{4}{3}ER^{\frac{1}{2}}\omega^{\frac{3}{2}},$$

(6) $$A_{e}=\pi R\omega .$$

when the interference equals the critical interference (*ω* = *ω_c_*), their critical values can be expressed as

(7) $$F_{c}=\frac{4}{3}ER^{\frac{1}{2}}\omega_{c}{}^{\frac{3}{2}},$$

(8) $$A_{c}=\pi R\omega_{c}.$$

These critical values predict the particular contact parameters at the inception of elastic–plastic deformation. Therefore, they are selected to nondimensionalize the results in all models.

## 3. Finite Element Model

**Figure 3:**
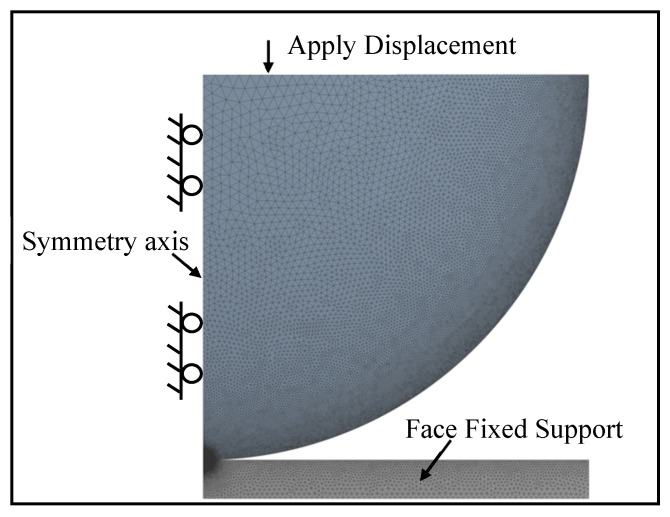
Finite element mesh and boundary conditions.

The finite element model presently used in this paper is similar to the finite element model described by the JG model [33], SM model [38,39], and SC model [41]. The commercial program ANSYS18.2-Workbench was used for modeling and analyzing the contact behavior of a deformable hemisphere pressed by a rigid flat. Due to its axial symmetry, a 2-D model was selected. The hemisphere was modeled by a quarter of a circle (*R* = 1 mm), while a half-space represented the rigid flat surface. The boundary load was applied, as shown in Figure 3. The nodes of a half-space were fixed in all directions to model the rigid flat. The nodes on the symmetry axis of the hemisphere were fixed in the radial direction to model a half-sphere. The tangent modulus and the friction coefficient were assumed as zero within ANSYS. The von Mises yield criterion was used to define material yield. The present analysis covers a wide range of material properties [48]. As shown in Table 2 and Table 3, the analyzed material properties were divided into two groups. Specifically, the property ranges of the 79.4 ≤ *E*/*Y* ≤ 800 and 0.2 ≤ *ν* ≤ 0.45 were considered. The half-sphere and half-space by PLANE 183 triangle elements were discretized in such a way that there were many elements near the contact edge, as shown in Figure 3. The mesh far from the contact edge became coarser to improve computing speed. The half-sphere and half-space total number of elements was 37,391 and 15,028, respectively. Additionally, their total number of nodes were 75,662, and 33,164, respectively. For the half-sphere, the JG model used a constant mesh of 11,101 elements, the KE model used a maximum of 2944 nodes in total, and the SM model used 9933 elements in total for their analysis. By comparison, the present model mesh was better. There are 2696 two-dimensional three-node surface contact elements, designated as CONTA172 and TARGE169 in ANSYS, to detect the contact behavior of a deformable hemisphere pressed by a rigid flat. The uniform displacement was applied to the bottom section of the hemisphere, and then the contact force was obtained by extracting the reaction force of the node at its bottom. The contact area was calculated by the contact radius, which was obtained by finding the contacting edge. The results compare well with the Hertz elastic solution at *ω* ≤ *ω_c_*. The error between them is less than 2%. In addition, to ensure mesh convergence, the mesh density was iterated twice until the result of each iteration changed by no more than 1%. Due to the material and geometric nonlinearity, the Augmented Lagrange algorithm was adopted, and a large deflection was activated. Additionally, multi-step loading was used to guarantee the solution convergence. The maximum number of substeps in the calculation for very large interference was 15,000. In order to obtain a generalized model, all contact parameters were in dimensionless form, i.e., *F*/*F_c_*, *ω*/*ω_c_*, *p*/*Y*, and *A*/*A_c_*. 

## 4. Results and Discussion

### 4.1. Contact Load

**Figure 4:**
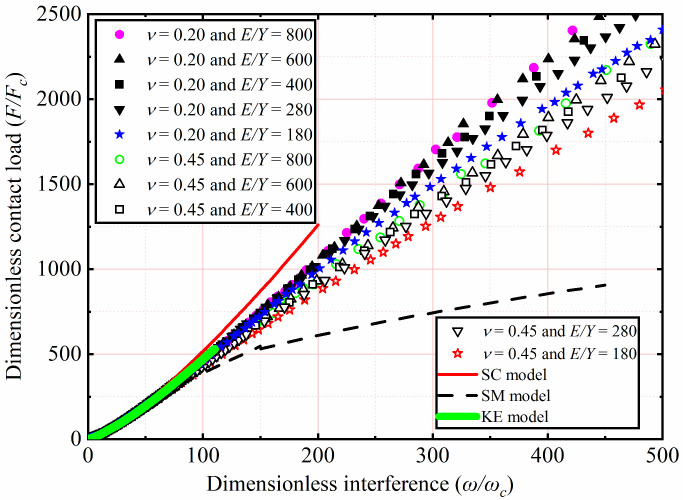
*F/F_c_* as a function of **ω*/*ω_c_** for different *E* and *ν*.

**Figure 5:**
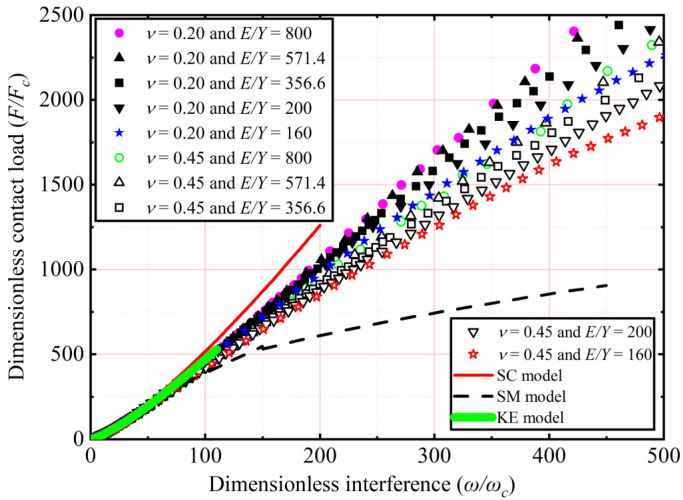
*F*/*F_c_* as a function of *ω*/*ω_c_* for different *Y*, *ν*, and *E*/*Y* > 133.3.

The effects of *E* and *ν* shown in Table 2 on *F*/*F_c_* as a function of *ω*/*ω_c_* were analyzed. Here *Y* was a constant value equal to 0.25 GPa, while *ν* were 0.2, 0.3, 0.4, and 0.45. With *E* ranging from 45 to 200 GPa, corresponding the *E*/*Y* ratios ranging from 180 to 800, respectively. Here only the results of *ν* = 0.2 and 0.45 for *E*/*Y* = 180, 280, 400, 600, and 800 were selected and plotted in Figure 4. *F*/*F_c_* increases with an increasing *ω*/*ω_c_*. For 1< *ω*/*ω_c_* ≤ 110, the results indicate the similarity with the KE model, *F*/*F_c_* is independent of *E* and *ν* of the material. For 110 < *ω*/*ω_c_* ≤ 500, *F*/*F_c_* decreased with a decreasing *E*. Moreover, it is clear from Figure 4 that the SM model underestimates *F*/*F_c_*. The reason of this discrepancy is that it is valid only for the *E*/*Y* ratio of 79.4. It is clear from Figure 5 that with the increase of *ω*/*ω_c_*, the present result is lower than *F*/*F_c_* predicted by the SC model. This may be the SC model analyzed the effect of strain hardening of materials on the elastic–plastic contact behavior, while the strain hardening effect is ignored in the present work. As **ω*/*ω_c_** increases, as shown in Figure 4, *F*/*F_c_* is also affected by *ν*. Additionally, for the fixed *E*/*Y*, the lower the *ν*, the higher *F*/*F_c_* is. 

The effects of *Y* and *ν* shown in Table 3 on *F*/*F_c_* as a function of *ω*/*ω_c_* are analyzed. Here *E* is a constant value equal to 200 GPa, while *ν* ranges from 0.2 to 0.45. With *Y* ranging from 1.25 to 0.25 GPa, corresponding to the *E*/*Y* ratios ranging from 160 to 800, which are all greater than 133.3, respectively. Here only the results of *ν* = 0.2 and 0.45 for *E*/*Y* = 160, 200, 356.6, 571.4, and 800 are selected and plotted in Figure 5. In the present interference domain, the SM model distinctly underestimates *F*/*F_c_*. For 1< *ω*/*ω_c_* ≤ 110, the results indicate that the similarity with the KE model, *F*/*F_c_* is independent of the *Y* and *ν* of the material. For 110 < *ω*/*ω_c_* ≤ 500, the present results show that the value of *F*/*F_c_* decreases with an increasing *Y*. In addition, as *ω*/*ω_c_* increases for the material with a lower *ν*, the higher the *F*/*F_c_* is for the fixed *E*/*Y*. In this work, the curve fitting of simulation data is carried out. For 133.3 < *E*/*Y* ≤ 800, 0.2 ≤ *ν* ≤ 0.45, and 1 < *ω*/*ω_c_* ≤ 500, the empirical expressions of *F*/*F_c_* as a function of *ω*/*ω_c_* is presented in this paper as follows:

(9) $$1\leq \frac{\omega }{\omega_{c}}\leq 10\begin{array}{cc} ; & \frac{F}{F_{c}}={\left(\frac{\omega }{\omega_{c}}\right)}^{m} \end{array},$$

(10) $$10\leq \frac{\omega }{\omega_{c}}\leq 500\begin{array}{cc} ; & \frac{F}{F_{c}}=n{\left(\frac{\omega }{\omega_{c}}\right)}^{q} \end{array}.$$

where *m*, *n*, and *q* are shown in Table 4. 

**Figure 6:**
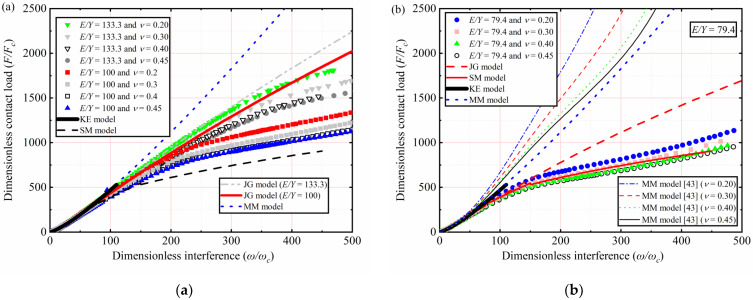
*F*/*F_c_* as a function of *ω*/*ω_c_* for different *Y*, *ν*, and *E*/*Y* ≤ 133.3: (**a**) *E*/*Y* = 133.3 and *E*/*Y* = 100; (**b**) *E*/*Y* = 79.4.

When *E*/*Y* is less than 133.3, *F*/*F_c_* as a function of *ω*/*ω_c_* for different *Y* and *ν* values is plotted in Figure 6. As shown in Table 3, *E* is 200 GPa, with *ν* = 0.2, 0.3, 0.4, and 0.45. With *Y* ranging from 2.52 to 1.5 GPa, corresponding to the *E*/*Y* ratio ranging from 79.4 to and 133.3, respectively. 

The results of *ν* = 0.2, 0.3, 0.4, and 0.45 for *E*/*Y* = 100 and 133.3 are selected and plotted in Figure 6a. *F*/*F_c_* increases with an increasing *ω*/*ω_c_*. For 1 < *ω*/*ω_c_* ≤ 110, the present trend is like the KE model for all models. However, *F*/*F_c_* is overestimated by the KE model for higher *Y* values and the interference near 110*ω_c_*. For 110 < *ω*/*ω_c_* ≤ 500, the present prediction results differ significantly from those of the JG model and MM model. Additionally, as *ω*/*ω_c_* increases, the lower the *E*/*Y*, the higher the deviation. At the value of *ω*/*ω_c_* equals 500, *F*/*F_c_* deviation between present work and JG model is 15.82% when the *E*/*Y* ratio is 133.3 and 46.4% when the *E*/*Y* ratio is 100. In the present interference domain, MM model underestimates *F*/*F_c_*. 

The results of *ν* = 0.2, 0.3, 0.4, and 0.45 for *E*/*Y* = 79.4 are selected and plotted in Figure 6b. For 1 < *ω*/*ω_c_* ≤ 110, the present trend is similar to the KE model. For 110 < *ω*/*ω_c_* ≤ 500, the JG model overestimates *F*/*F_c_* in the present interference domain. At the value of *ω*/*ω_c_* equals 500, *F*/*F_c_* deviation between the present work and the JG model is 49.8% at the *E*/*Y* of 79.4. The obtained result is only similar to the SM model, which is valid only when the *E*/*Y* ratio is 79.4 and *ν* is 0.3. However, in present work, *ν* = 0.2, 0.3, 0.4, and 0.45 are further considered when the *E*/*Y* ratio is 79.4. It can be seen that *F*/*F_c_* is affected by *ν* and that the lower the *ν*, the higher the *F*/*F_c_* is with the increased *ω*/*ω_c_*. In this work, the curve fitting of simulation data is carried out. For 79.4 ≤ *E*/*Y* ≤ 133.3, 0.2 ≤ *ν* ≤ 0.45, and 1 < *ω*/*ω_c_* ≤ 500, the empirical expressions of *F*/*F_c_* as a function of *ω*/*ω_c_* are presented in this paper as follows:

(11) $$79.4\leq \frac{E}{Y}\leq 133.3\begin{array}{cc} , & \frac{F}{F_{c}}=m{\left(\frac{\omega }{\omega_{c}}\right)}^{n} \end{array}.$$

where *m* and *n* are shown in Table 5.

### 4.2. Contact Area

**Figure 7:**
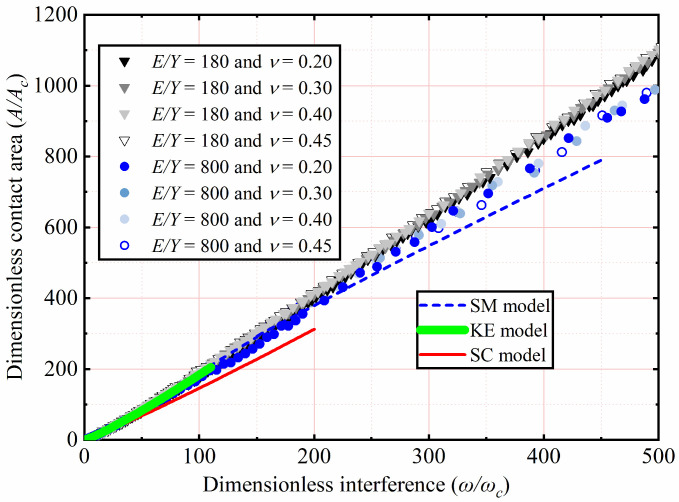
*A*/*A_c_* as a function of *ω*/*ω_c_* for different *E* and *ν*.

The effects of *E* and *ν* shown in Table 2 on *A*/*A_c_* as a function of *ω*/*ω_c_* are analyzed. Here *Y* is a constant value equal to 0.25 GPa, with *ν* ranging from 0.2 to 0.45. With *E* ranging from 45 to 200 GPa, corresponding the *E*/*Y* ratios ranging from 180 to 800, respectively. Here only the results of *ν* = 0.2, 0.3, 0.4, and 0.45 for *E*/*Y* = 180 and 800 are selected and plotted in Figure 7. For 1 < *ω*/*ω_c_* ≤ 110, the results indicate the similarity with the KE model, and *A*/*A_c_* is independent of *E* and *ν*. For 110 < *ω*/*ω_c_* ≤ 500, *A*/*A_c_* increases with an increasing *ω*/*ω_c_*. In addition, the results show that *E* and *ν* have little effect on *A*/*A_c_*. It is clear from Figure 7 that the SM model underestimates *A*/*A_c_* as *ω*/*ω_c_* increases, especially for large interference. The reason of this discrepancy is that the SM model is valid only for an *E*/*Y* ratio of 79.4. The present result is slightly higher than the *A*/*A_c_* predicted by the SC model with an increasing **ω*/*ω_c_**. The SC model analyzed the effect of tangential modulus on the elastic–plastic contact behavior, while the strain hardening effect is ignored in the present study. Compared with the predicted results of the SC model, it can be observed that tangential modulus has a great effect on the elastic–plastic contact behavior. In view of the shortcomings of present study, we will consider the effect of tangential modulus on hemispheric elastic–plastic contact behavior under large interference in future work. 

**Figure 8:**
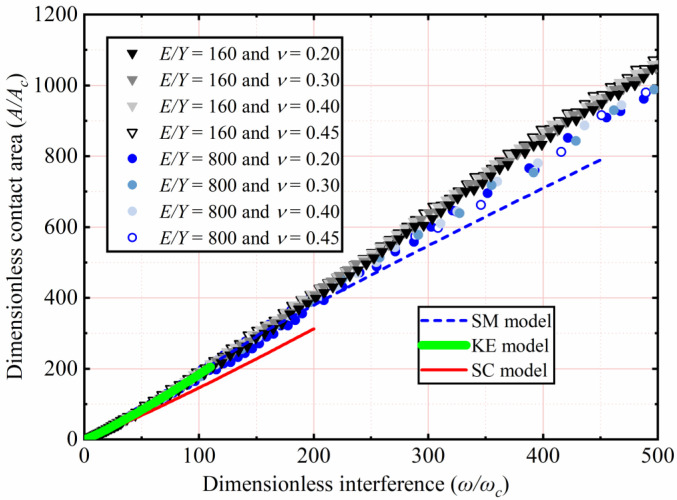
*A*/*A_c_* as a function of *ω*/*ω_c_* for different *Y*, *ν*, and *E*/*Y* > 133.3.

The effects of *Y* and *ν*, as shown in Table 3, on *A*/*A_c_* as a function of *ω*/*ω_c_* are analyzed. Here *E* is 200 GPa, with *ν* ranging from 0.2 to 0.45. With *Y* ranging from 1.25 to 0.25 GPa, corresponding the *E*/*Y* ratios ranging from 160 to 800, respectively. Here only the results of *ν* = 0.2, 0.3, 0.4, and 0.45 for *E*/*Y* = 160 and 800 are selected and plotted in Figure 8. *A*/*A_c_* increases with an increasing *ω*/*ω_c_*. For 1 < *ω*/*ω_c_* ≤ 110, the results indicate the similarity with the KE model, *A*/*A_c_* is independent of *Y* and *ν*. For 110 < *ω*/*ω_c_* ≤ 500, *Y* and *ν* have little effect on *A*/*A_c_*. Since the SM model is only suitable for *E*/*Y* = 79.4, *A*/*A_c_* is underestimated in the present interference domain. 

In Figure 7 and Figure 8, the effects of *E*, *ν*, and *Y* on *A/A_c_* are comprehensively considered under an *E*/*Y* greater than 133.3. The results show that the *E*/*Y* ratio affects *A*/*A_c_*. As the *ω*/*ω_c_* increases, the lower the *E*/*Y*, the higher the *A*/*A_c_* is. *ν* has little effect on *A*/*A_c_*. In this work, the curve fitting of simulation data is carried out. For 133.3 < *E*/*Y* ≤ 800, 0.2 ≤ *ν* ≤ 0.45, and 1 < *ω*/*ω_c_* ≤ 500, the empirical expressions of *A*/*A_c_* as a function of *ω*/*ω_c_* is presented in this paper as follows:

(12) $$133.3\leq \frac{E}{Y}\leq 800\begin{array}{cc} ; & \frac{A}{A_{c}}=m{\left(\frac{\omega }{\omega_{c}}\right)}^{n} \end{array}.$$

where *m* and *n* are shown in Table 6.

**Figure 9:**
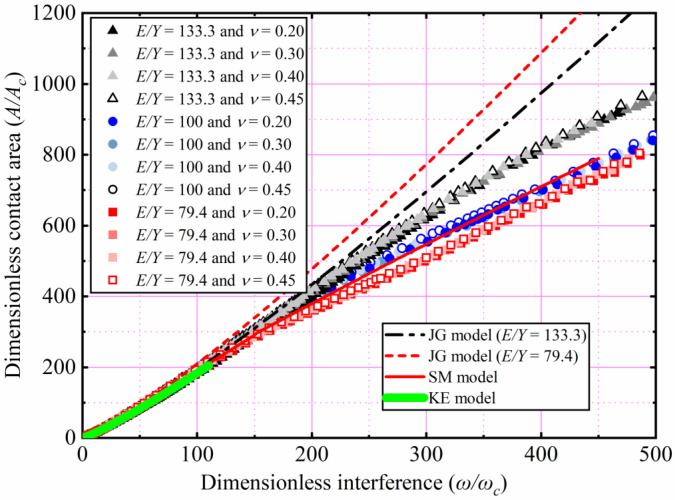
*A*/*A_c_* as a function of *ω*/*ω_c_* for different *Y*, *ν* and *E*/*Y* ≤ 133.3.

When the ratio of *E*/*Y* is less than 133.3, *A*/*A_c_* as a function of *ω*/*ω_c_* for different *Y* and *ν* values is plotted in Figure 9. Here *E* is a constant value equal to 200 GPa, with *ν* ranging from 0.2 to 0.45. With *Y* being 2.52, 2, and 1.5 GPa, corresponding the *E*/*Y* ratio is 79.4, 100, and 133.3, respectively. *ω*/*ω_c_* ranges from 1 to 500. At small interferences, the dependence of *A*/*A_c_* on *Y* and *ν* is weak, which is similar to the prediction of KE model. However, with an increasing *ω*/*ω_c_*, the influence of *Y* on *A*/*A_c_* is strong. It is clear from Figure 9 that the present *A*/*A_c_* is consistent with that predicted by the SM model for *E*/*Y* = 79.4. However, *A*/*A_c_* is underestimated by the SM model when the *E*/*Y* is greater than 79.4. It can also be seen that in the present *ω*/*ω_c_* domain, the JG model overestimates *A*/*A_c_*. At a *ω*/*ω_c_* value of 500, *A*/*A_c_* deviation between the present work and JG model is 70.73% when *E*/*Y* is 133.3 and 25.1% when *E*/*Y* is 79.4. When *E*/*Y* is greater than 133.3, the error of the JG model is slightly less than that of other models compared with the present predicted results. To overcome the drawbacks of previous models, the curve fitting of the simulation data is carried out in this work. For 79.4 ≤ *E*/*Y* ≤ 133.3, 0.2 ≤ *ν* ≤ 0.45, and 1 < *ω*/*ω_c_* ≤ 500, the empirical formulas for predicting the contact area are presented in this paper as follows:

(13) $$\begin{array}{cc} 79.4\leq \frac{E}{Y}\leq 133.3, & \end{array}\frac{A}{A_{c}}=m{\left(\frac{\omega }{\omega_{c}}\right)}^{n}.$$

where *m* and *n* are shown in Table 7.

### 4.3. Contact Pressure

**Figure 10:**
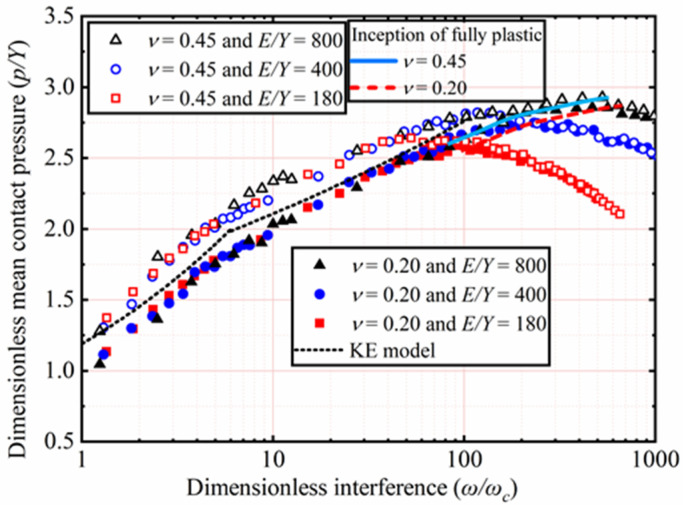
*p/Y* as a function of **ω*/*ω_c_** for different *E* and *ν*.

The effects of *E* and *ν* shown in Table 2 on *p*/*Y* as a function of *ω*/*ω_c_* are analyzed. Here, *Y* is a constant value equal to 0.25 GPa, with *ν* ranging from 0.2 to 0.45. With *E* ranging from 45 to 200 GPa, corresponding the *E*/*Y* ratios ranging from 180 to 800, respectively. Here, only the results of *ν* = 0.2 and 0.45 for *E*/*Y* = 180, 400, and 800 are selected and plotted in Figure 10. The KE model considered that *p/Y* reaching the peak could be marked the contact state transition from the elastic–plastic to the fully plastic and that the corresponding **ω*/*ω_c_** is a constant value equal to 110. Through this, the elastic–plastic range (1 ≤ **ω*/*ω_c_** ≤ 110) is proposed by the KE model. However, this tendency is not observed in the present work. It is obvious that **ω*/*ω_c_** corresponding to the peak value of *p/Y* is not fixed but is dependent on *E* and *ν* in all cases. It is clear from Figure 10 that the peak value of *p/Y* also increases with an increasing *E*. Additionally, the value of *ν* increases, the peak value of *p/Y* also increases under constant *E*/*Y*. By comparison, the effects of *ν* are less than that of *E* on the peak value of *p/Y*. 

After *p/Y* reaches its peak value, namely the fully plastic range, it is no longer affected by *ν*, as shown in Figure 10. In the fully plastic range, *p/Y* decreases with an increasing **ω*/*ω_c_**. Moreover, the results also show that this trend is more evident with the lower *E* value, which is the same as the observational results of the SC model. Interestingly, the results obtained show that before *p/Y* reaches its peak in all cases, namely the elastic–plastic range, the higher ν, the higher *p/Y* is. 

**Figure 11:**
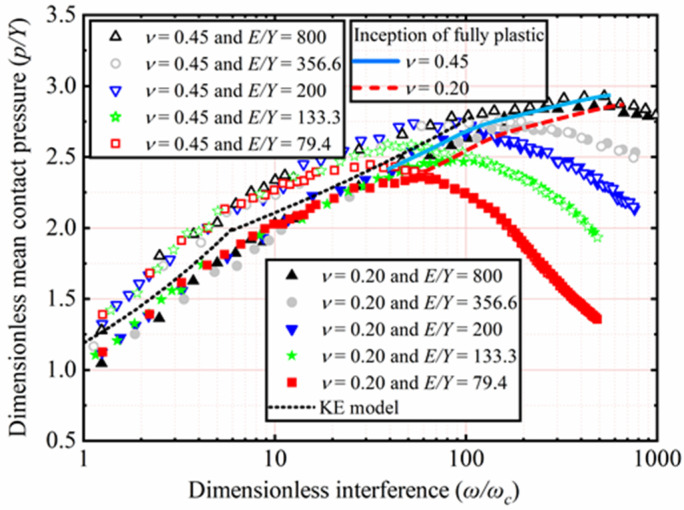
*p/Y* as a function of **ω*/*ω_c_** for different *Y* and *ν*.

The effects of *Y* and *ν*, as shown in Table 3, on *p/Y* as a function of **ω*/*ω_c_** are analyzed. Here *E* is a constant value equal to 200 GPa, with *ν* ranging from 0.2 to 0.45. With *Y* ranging from 2.52 to 0.25 GPa, corresponding the *E*/*Y* ratios ranging from 79.4 to 800, respectively. Here only the results of *ν* = 0.2 and 0.45 for *E*/*Y* = 79.4, 133.3, 200, 356.6, and 800 are selected and plotted in Figure 11. In the elastic–plastic range, *p/Y* increases with an increasing **ω*/*ω_c_**. Additionally, the higher *ν*, the higher *p/Y* is. *p/Y* decreases with an increasing **ω*/*ω_c_** in the plastic range. The results show that this trend is more evident with the higher *Y* value. Moreover, *ν* has no longer has an effect on *p/Y* in the fully plastic range. 

The present work has observed that in all cases, **ω*/*ω_c_** corresponding to the peak value of *p/Y* is not limited to a specific value of 110 predicted by the KE model but depends on *Y* and *ν*. It is clear from Figure 11 that the peak value of *p/Y* also increases when *Y* decreases. Moreover, as *ν* increases, the peak value of *p/Y* also increases under constant *E*/*Y*. By comparison, the effects of *ν* are less than *Y* for the peak value of *p/Y*. 

### 4.4. Elastic–Plastic Range

**Figure 12:**
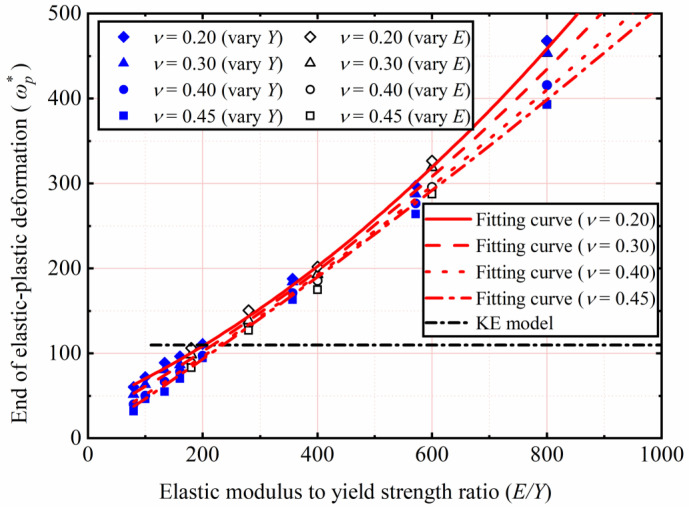
$\omega_{p}^{*}$ as a function of *E*/*Y*.

Figure 12 shows the end of the elastic–plastic deformation ($\omega_{p}^{*}$) as a function of *E*/*Y*. In addition, comparisons with the KE model are also depicted. The KE model suggested that *p/Y* reaches the peak and marks the transition from the elastic–plastic to the fully plastic deformation regime, and the corresponding $\omega_{p}^{*}$ is the constant value equal to 110. The method of using the maximum value of *p/Y* to find the end of the elastic–plastic deformation regime can also be found in Kogut et al. [31,36]. The present results show that the value of $\omega_{p}^{*}$ increases with an increasing of *E*/*Y* and is not the constant value equal to 110 predicted by the KE model. For all of *E*/*Y*, the higher the value of *ν*, the lower value of $\omega_{p}^{*}$. The present results also show that the value of $\omega_{p}^{*}$. is about 110 at *E*/*Y* = 200, which is consistent with the predicted result of the KE model. Moreover, when the *E*/*Y* is less than 200, the KE model overestimates the value of $\omega_{p}^{*}$. Therefore, to estimate the value of $\omega_{p}^{*}$ and predict the end of the elastic–plastic range, the empirical formula is proposed in this work as follows:

(14) $$\omega_{p}^{*}=\left[4.631\times 10^{-4}-\left(8.8827\times 10^{-4}\right)\nu \right]{\left(\frac{E}{Y}\right)}^{2}+\left(0.18+0.59\nu \right)\frac{E}{Y}-143.53\nu +66.42.$$

As mentioned above, the corresponding *ω_c_* at the inception of the elastic–plastic deformation regime can be determined by using Equation (4). Additionally, the corresponding $\omega_{p}^{*}$ at the end of elastic–plastic deformation regime can be determined by using Equation (14). Furthermore, to estimate the contact parameters of the elastic–plastic range, the empirical formula is proposed in this work as follows:

(15) $$F_{ep}^{*}=m{\left(\frac{\omega }{\omega_{c}}\right)}^{n},$$

(16) $$A_{ep}^{*}=q{\left(\frac{\omega }{\omega_{c}}\right)}^{t}.$$

where *m, n, q,* and *t* are shown in Table 8.

**Figure 13:**
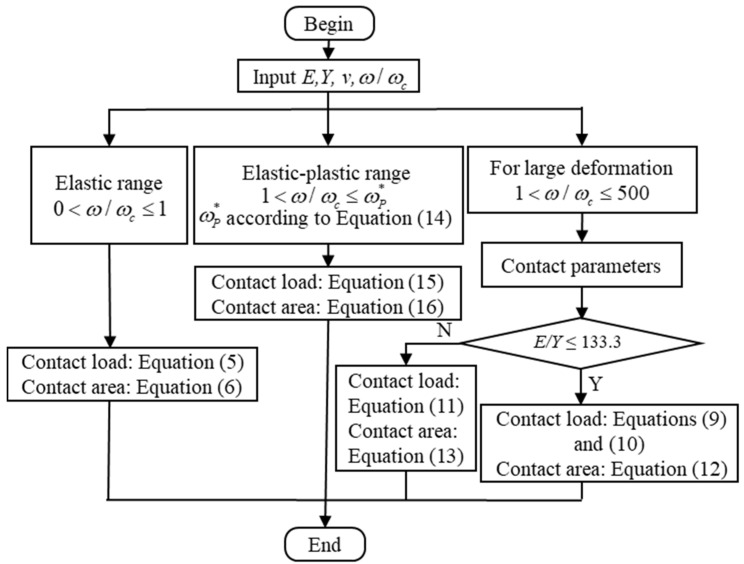
Flowchart for predicting the contact parameters.

In order to more conveniently use the elastic–plastic constitutive relation presented in this paper, a flowchart for predicting the contact parameters in the purely elastic, elastic–plastic, and fully plastic ranges is shown in Figure 13. First, for the purely elastic contact stage, the contact parameters are calculated according to Equations (5) and (6). Secondly, for the elastic–plastic range, the contact parameters are calculated according to Equations (15) and (16). Additionally, and lastly, for large deformations, it includes the elastic–plastic and fully plastic range. If 0.2 ≤ *ν* ≤ 0.45 and 79.4 ≤ *E*/*Y* ≤ 133.3, the contact parameters are calculated according to Equations (11) and (13), otherwise according to the Equations (9)–(12). 

### 4.5. Comparison with Experimental Results

To verify the rationality of the present model, the present predicted contact load and area are compared with Ovcharenko et al. [28] and Jamari and Schipper [27] experimental data. 

**Figure 14:**
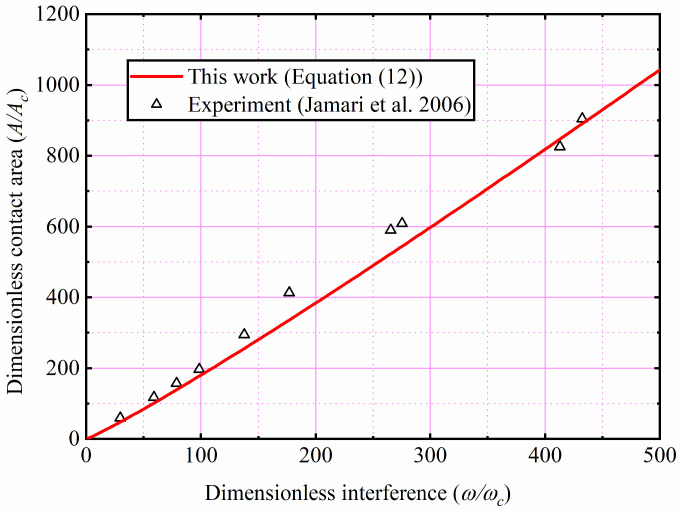
Comparison with experimental result for the copper [27].

Jamari and Schipper [27] experimentally measured the relationship between the contact parameters of the copper sphere and the SiC ceramic flat. In their experiment, the material parameters of the copper sphere are *E* = 120 GPa, *R* = 1.5 mm, *ν* = 0.35, *H* = 1.2 GPa, and *E*/*Y* = 280, respectively. As their material parameter is *E*/*Y* = 280, Jamari and Schipper’s experimental results were compared with the present prediction formula of the Equation (12) and the results are shown in Figure 14. The present prediction results are in good agreement with the experimental ones, which verifies the accuracy of the present work. 

**Figure 15:**
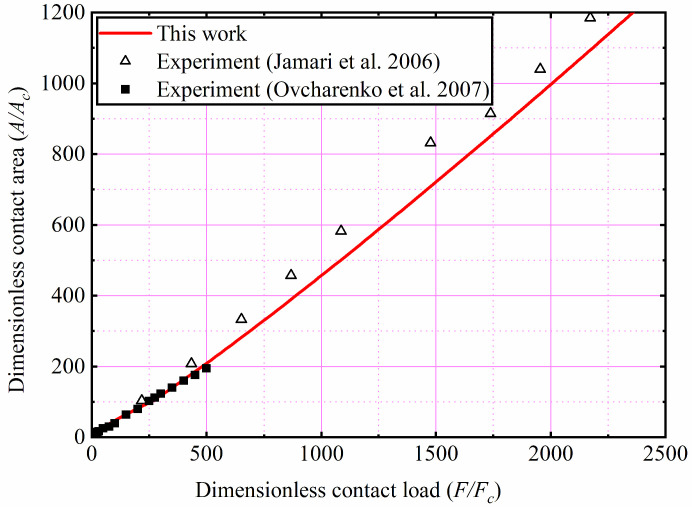
Comparison with experimental results for the stainless-steel and copper [27,28].

Ovcharenko et al. [28] experimentally measured the relationship between the contact load and the area of the stainless-steel and copper spheres in contact with the Sapphire flat. As shown in the Figure 15. the present predicted results are compared with the Ovcharenko et al. experimental results for the copper and stainless-steel. Compared with the Jamari and Schipper experimental study, Ovcharenko et al. only analyzed the contact area under small loads through their experiments. It is clear from Figure 15 that the present model is in good agreement with the experimental results and the error is less than 10%, indicating that the proposed model has a high accuracy. This discrepancy may be due to the fact that the SiC ceramic flat in the experiment is not as rigid as assumed in the theoretical model, leading to some error between the experimental and theoretical results. 

## 5. Conclusions

A flattening contact behavior of an elastic–perfectly plastic hemisphere against a rigid flat was researched by using FEM. The present analysis addresses the effects of Poisson’s ratio, elastic modulus, and yield strength on the contact behavior. The main conclusions can be drawn as follows:

(1) The present study considers a large range of dimensionless interference from 1 to 500. A new elastic–plastic constitutive model is proposed to predict the contact area and load based on the curve fitting the finite analyses results. Compared with previous models and experiments, the rationality of the present model is verified.
(2) *p/Y* is mainly affected by the *ν* in the elastic–plastic range, and the higher the *ν*, the higher *p/Y*. However, this influence disappears in the fully plastic range. The maximum of *p/Y* is not a constant value for the different *E*, *Y*, and *ν*. The higher *E*/*Y*, the higher the maximum of *p/Y* is. Moreover, the higher *ν*, the higher the maximum value of *p/Y* is when *E*/*Y* is constant. However, the effects of *ν* are less than that of *E* and *Y* for the maximum value of *p/Y*. In the plastic range, *p/Y* decreases with increasing interference. Additionally, the lower *E*/*Y*, the more noticeable this trend is.
(3) The boundaries between the elastic, elastic–plastic, and fully plastic deformation regimes are determined according to the interference, maximum mean contact pressure, Poisson’s ratio, and the elastic modulus to yield strength ratio. When the interference is small, the contact state is purely elastic, and the contact parameters can be calculated according to the Hertz formula. When the interference increases, the contact state changes from the purely elastic to the elastic–plastic. The present work shows that the JG model can more reasonably determine the inception of elastic–plastic deformation regime. The end of the elastic–plastic deformation regime is defined according to the interference corresponding to the maximum contact pressure. New dimensionless constitutive relationships are proposed to predict the contact parameters in the elastic–plastic range.

## Figures and Tables

**Table 1:** Summary of maximum contact pressure (*p*_0_).

Models	*p*_0_	*K* or *H*	Yield Strength Coefficient (*C*)
GW model	*p*_0_ = 0.6*H* or *p*_0_ = *CY*	*H* = 2.8*Y*	1.68
CEB model	*p*_0_ = *KH* or *p*_0_ = *CY*	*K* = 0.41*ν* + 0.454 *H* = 2.8*Y*	1.148*ν* + 1.2712
Green model	*p*_0_ = *CY*	-	0.54373*ν^2^* + 0.8782*ν* + 1.30075
JG model	*p*_0_ = *CY*	-	1.295 exp(0.736*ν*)
LL model	*p*_0_ = *KH* or *p*_0_ = *CY*	*K* = 0.1943*ν*^2^ + 0.3141*ν* + 0.4645 *H* = 2.8*Y*	0.54404*ν^2^* + 0.87948*ν* + 1.3006
BK model	*p*_0_ = *CY*	-	1.256*ν* + 1.234

**Table 2:** Material properties in the first group.

No.1	*E* (GPa)	*Y* (GPa)	*E*/*Y*	*ω*/*ω_c_*	*R* (mm)	*ν*
1	45	0.25	180	500	1	0.2, 0.3, 0.4, 0.45
2	70	0.25	280			
3	100	0.25	400			
4	150	0.25	600			
5	200	0.25	800			

**Table 3:** Material properties in the second group.

No.2	*E* (GPa)	*Y* (GPa)	*E*/*Y*	**ω*/*ω_c_**	*R* (mm)	*ν*
1	200	2.52	79.4	500	1	0.2, 0.3, 0.4, 0.45
2	200	2	100			
3	200	1.5	133.3			
4	200	1.25	160			
5	200	1	200			
6	200	0.56	356.6			
7	200	0.35	571.4			
8	200	0.25	800			

**Table 4:** Parameters in Equations (9) and (10).

*E*/*Y*	*m*	*n*	*q*
133.3 < *E*/*Y* < 200	1.756 − (1.5 × 10^−4^) (*E*/*Y*) − 0.1*ν*	5.51	0.982 − (1 × 10^−4^) (*E*/*Y*) − 0.1*ν*
200 ≤ *E*/*Y* ≤ 800	1.610 + (2.0 × 10^−5^) (*E*/*Y*) − 0.1*ν*	3.52	1.094 − (2 × 10^−5^) (*E*/*Y*) − 0.1*ν*

**Table 5:** Parameters in Equation (11).

	1 < *ω*/*ω_c_* ≤ 5	5 < *ω*/*ω_c_* ≤ 90	90 < *ω*/*ω_c_* ≤ 5.4 (*E*/*Y*)	5.4 (*E*/*Y*) < *ω*/*ω_c_* ≤ 500
*m*	1.00	1.70	0.024 (*E*/*Y*) − 3*ν* + 7.3	0.65 (*E*/*Y*) − 30.5*ν* + 7.74
*n*	1.55	1.20	0.84	0.48

**Table 6:** Parameters in Equation (12).

	1 < *ω*/*ω_c_* ≤ 10	10 < *ω*/*ω_c_* ≤ 500
*m*	1.02	1.3
*n*	1.171 − (2 × 10^−5^) (*E*/*Y*)	1.096 − (2 × 10^−5^) (*E*/*Y*)

**Table 7:** Parameters in Equation (13).

	1 < *ω*/*ω_c_* ≤ 5	5 < *ω*/*ω_c_* ≤ 90	90 < *ω*/*ω_c_* ≤ 5.4 (*E*/*Y*)	5.4 (*E*/*Y*) < *ω*/*ω_c_* ≤ 500
*m*	0.998	0.997	0.0073 (*E*/*Y*) + 2.13	0.0093 (*E*/*Y*) + 2.26
*n*	1.14	1.15	0.923	0.903

**Table 8:** Parameters in Equations (15) and (16).

	*m*	*n*	*q*	*t*
1 < *ω*/*ω_c_* ≤ 5	1	1.55	0.997	1.14
$5\;<\;\omega /\omega_{c}\;\leq \;\omega_{p}^{*}$	1.89	1.206 − 0.1*ν*	1.1	1.10

## Data Availability

Not applicable.

## Nomenclature

a	Contact radius
*p*	Mean contact pressure
*p*_0_	Maximum Hertzian pressure
*A*	Contact area
*C*	Critical yield stress coefficient
*E*	Equivalent elastic modulus
*F*	Contact load
$\omega_{p}^{*}$	Critical interference value at the inception fully plastic
*R*	The radius of asperity
*Y*	Yield strength
*ν*	Poisson’s ratio
*ω*	Normal interference of asperity
Scripts	
1	The value of asperity
2	The value of rigid flat
*c*	Critical values at yielding inception
*p*	Critical values at the end of the elastic–plastic range
***	Dimensionless
*e*	Elastic range
*ep*	Elastic–plastic range
